# Comparing the effect of encapsulated and unencapsulated fennel extracts on the shelf life of minced common kilka (*Clupeonella cultriventris caspia*) and *Pseudomonas aeruginosa* inoculated in the mince

**DOI:** 10.1002/fsn3.275

**Published:** 2015-09-04

**Authors:** Roya Bagheri, Rabeeh Izadi Amoli, Nastaran Tabari Shahndasht, Seyed Rasoul Shahosseini

**Affiliations:** ^1^ Department of Food Science and TechnologyAyatollah Amoli BranchIslamic Azad UniversityAmolIran; ^2^ Department of MicrobiologyAyatollah Amoli BranchIslamic Azad UniversityAmolIran; ^3^ Student of Food ScienceAyatollah Amoli BranchIslamic Azad UniversityAmolIran; ^4^ Young Researchers ClubAyatollah Amoli BranchIslamic Azad UniversityAmolIran

**Keywords:** Encapsulation, fennel extract, fish mince, gum arabic, kilka

## Abstract

The quality of minced kilka (*Clupeonella cultriventris caspia*) with gum arabic encapsulated (0.3% and 0.5% w/w) and unencapsulated fennel extract (FE) (0.3% and 0.5% w/w) stored at 4°C was examined over a storage period of 15 days. The control and the treated fish samples were analyzed periodically for microbiological (total viable count [TVC] and total psychrotrophic count [TPC]) and chemical (peroxide value (PV) and total volatile nitrogen (TVB‐N)) parameters. Also the inhibitory effect of encapsulated and unencapsulated FE was evaluated against *Pseudomonas aeruginosa*, inoculated in minced kilka. According to the results, encapsulated FE samples showed the lowest amount of lipid oxidation and microbial deterioration during the storage period compared with the control and pure extract treatments. Although, the encapsulated FE at 0.5% showed drastic bacterial effect against *Pseudomonas aeruginosa* compared to others. Generally, gum arabic encapsulation could help to obtain higher antimicrobial and antioxidant activity in lower FE concentrations in minced fish.

## Introduction

Kilka is the most abundant fish in the Caspian Sea located in the north of Iran. Three species of Kilka are anchovy Kilka (*Clupeonella engrauliformis*), common Kilka (*C. cultriventris caspia*), and bigeye Kilka (*C. grimmi*) (Fazli et al. 2009). Common Kilka has been identified as one of the most important industrial and commercial fish in the Caspian Sea. A total catch of 24, 000 tons of common Kilka was reported by the Iranian Fisheries Organization for the year 2010 (Shilat 2010). Unfortunately, a small portion of the Kilka catch is used by human (4%) due to its small size, easy deterioration, impossibility of gutting right away after catching, difficulties in hygienic preservation, packaging, and supply and the rest is used as fish meal for poultry and in aquaculture (Khoshkhoo et al. 2010; Khanipour et al. 2014). Consequently, to increase the amount of consumption, it is proposed to have various fish products derived from it. Today, production of minced fish which are vastly consumed could be one way of increasing fish consumption (Asgharzadeh et al. 2006). It is an intermediately ready product for preparation of different highly acceptable ready to serve seafood products like frozen surimi, frozen mince block, extruded product, imitated products, etc. (Devi et al. 2013).

Spoilage of fish results from changes brought about by biological reactions such as oxidation of lipids, the activities of the fish muscular enzymes, and the metabolic activities of microorganisms. Minced fish is more susceptible for microbial spoilage than whole fish due to greater surface area of minced fish. Food‐borne infections are one of the most serious and costly public health concerns worldwide. Increasing demand for high‐quality fish products with extended shelf life has initiated the development of several innovative techniques to keep quality attributes as long as possible and yield safe products (Maftoonazad and Badii 2009).

Lightly preserved food products with natural additives have become popular due to the modern trends adopted by consumers toward the consumption of food and seafood free from pathogens, with minimally processed foodstuffs containing no chemical preservatives (Abdollahzadeh et al. 2014). Fennel (*Foeniculum vulgare*) is one of the most important medicinal and aromatic plants due to its estrogenic activities and applications as a carminative, diuretic, anti‐inflammatory, antimicrobial, and galactogogue; it is a substance which is used to increase the production of milk in humans and animals (Mahfouz 2007). Fennel is also used for its medicinal properties; it is nowadays cultivated for industrial uses such as cosmetic and pharmaceutical products (El Ouariachi et al. 2014). *Foeniculum vulgare* extract demonstrated bacteriostatic activity against the following bacteria using the agar diffusion and serial dilution method: *Bacillus subtilis*,* Escherichia coli*, and *Pseudomonas aeruginosa* (Bone and Mills 2013). It has shown antimicrobial (Gulfraz et al. 2008) antioxidant activity in various in vivo and in vitro experiments (Barros et al. 2009; El Ouariachi et al. 2014), its ability in fish preservation has not been studied.

However, plant extracts generally present some formulation problems, such as long‐term instability, low bioavailability, and a significant burst release. Therefore, there is a need for their protection. Encapsulation is proposed as a convenient method for that purpose (Rijo et al. 2014). It was already explained that, encapsulation can protect antioxidants from light and oxygen, and preserve their stability and activity to some extent (Belščak‐Cvitanović et al. 2011; Bojanaa et al. 2013). Some studies (Gortzi et al. 2007; Bojanaa et al. 2013) have also shown that encapsulation can improve antimicrobial and antioxidant activity of compounds and maintain the stability of them over prolonged periods of time.

Thus, this study was aimed to investigate the effect of encapsulated and unencapsulated fennel extract on the quality of minced common Kilka during storage at refrigeration condition (4 ± 1°C) and behavior of *Pseudomonas aeruginosa* inoculated to it.

## Materials and Methods

### Materials

The plant fennel (*Foeniculum vulgare*) was purchased from a local market and authenticated by the Medical Plant Incubator Center (Islamic Azad University, Ayatollah Amoli Branch, Amol, Iran). The solvent (ethanol) was added to powdered fennel in ratio of 1:10 and the resulting mixtures were shaken overnight to extract fennel's phenol compounds. After 24 h, the extracts were filtered through Whatman No. 42 filter paper to separate thyme particles. The solvents were completely evaporated in an oven at 40°C. Finally, they were placed in a refrigerator (Esmaeilzadeh Kenari et al. 2014). *Pseudomonas aeruginosa* was purchased from the Iranian Research Organization for Science and Technology (Persian Type Culture Collection (PTCC), Tehran, Iran). Gum arabic was obtained from Sigma‐Aldrich Chemical Co (St Louis, MO, U.S.). All other chemicals were of analytical grade.

### Encapsulation by spray drying

Encapsulation of the fennel extract with gum arabic was done according to the method described by Beristain (2001) with some modifications. Solutions of gum arabic were prepared by dissolving 300 g (w/w) of gum arabic powder in distilled water. The obtained solutions were heated at 60°C with constant stirring for 20 min, covered, and left overnight at room temperature. Fennel extract was added to the hydrated gum solution at extract: gum ratio of 1:4 w/w with respect to gum solids. The mixture was homogenized with an Ultra‐Turrax T50 homogenizer (IKA‐WERKE Works Inc., Wilmington, NC) at 7000 rpm for 15 min and fed into a Buchii Mini Spray Dryer model 190 (Buchii Laboratoriums‐Technik AG., Flawil, Switzerland). The drying condition inlet air temperature was 105°C and the outlet air temperature was 108°C with a feeding rate of 5.6 mL/min.

Encapsulated and unencapsulated fennel extract was used in the preparation of 0.3% and 0.5% (w/v) solution in distilled water of 4°C.

### Fish sample preparation and storage condition

Fifty live commercial‐sized Kilka with an average weight 7 g were purchased from a public market alive and transferred to the Caspian Sea Ecology Research Center in Iran in sealed and foamed polystyrene boxes containing flaked ice. Then the fishes were killed by slurry ice, washed by hand, and minced twice. Furthermore, a ranking test previously carried out comparing fish samples with encapsulated fennel at different concentrations showed significantly lower acceptability of the samples incorporating 0.75 or 1% encapsulated fennel when compared to the rest (0.5% or lower) (data not shown). After these results, the encapsulated fennel concentration of 0.3% and 0.5% was chosen as optimal for the following study of fish preservation, and then minced fish was randomly assigned into five batches (100 ± 10 g minced fish in each group). The first batch was prepared without fennel extract (control batch), four of the batches processed with unencapsulated and encapsulated fennel extract as following; batch 2 (0.3% unencapsulated RE), batch 3 (0.5% unencapsulated RE), batch 4 (0.3% encapsulated RE), batch 5 (0.5% encapsulated RE). For *Pseudomonas aeruginosa* analysis, minced samples were inoculated with 1 × 10^4^ CFU/g of *Pseudomonas aeruginosa*. Then, the encapsulated and unencapsulated fennel extract were added according to the above‐mentioned treatments. After packaging, all samples in the polyethylene dishes were covered with a cellophane blanket, they were stored at 4 ± 1 °C for subsequent quality assessment. Chemical and microbiological analyses were performed at 3 day intervals to determine the overall quality of the minces.

### Chemical analysis

The peroxide value (PV) of the samples was determined in the total lipid extracts according to the method of Pearson (Egan et al. 1997). Results were expressed in meq oxygen kg‐1 lipids.

The total volatile basic nitrogen (TVB‐N) of the samples was determined by the microdiffusion method as described by Goulas and Kontominas (2005). Results were expressed in mg N/100 g of fish and performed in triplicate.

### Microbiological analysis

Bacteriological counts were determined by placing a 10 g of the mince sample in 90 mL of physiological serum, and homogenizing with a stomacher. The total viable count (TVC) and total psychrotrophic count (TPC) of the samples were determined by the pour plate method, using plate count agar (PCA, Merk, Darmstadt, Germany). The inoculated plates were incubated at 37°C for 2 days for TVCs, and at 10 °C for 7 days for psychrotrophilic counts)AOAC 2005(.

For *Pseudomonas aeruginosa* enumerations, 0.1 mL from 1:10 prepared serial dilutions (0.1% physiological solution) of fish mince homogenates was spread onto the surface of solid media. *Pseudomonas* was determined on Pseudomonas Isolation agar (PIA, Oxoid, UK) after incubation at 48 h at 35 °C. This medium is selective and formulated to enhance the formation of blue or blue‐green pyocyanin pigment by *P. aeruginosa* (Pavelková et al. 2014). All counts were expressed as log colony‐forming units (CFU/g) and performed in triplicate (Ibrahim Sallam ).

### Statistical analysis

One‐way ANOVA was used and mean comparison was performed by Duncan's new multiple range test. Statistical analysis was prepared using the SPSS statistical software, (release 18.0) for Windows (SPSS Inc. Chicago, IL). All data are presented as mean ±SD. Significant differences were considered at the 95% confidence level (P < 0.05).

## Results and discussion

### Changes in peroxide value (PV) content

**Figure 1:**
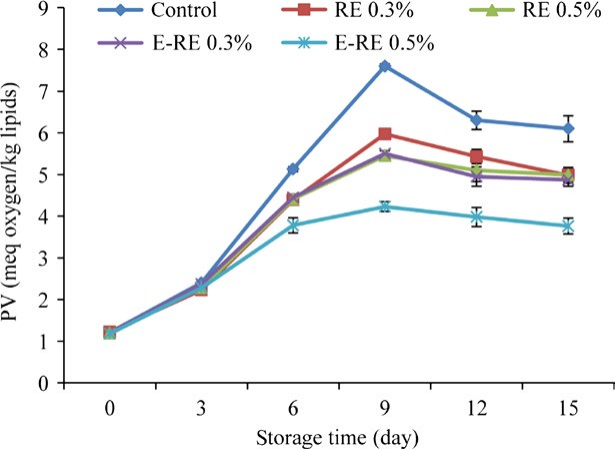
Changes in the peroxide value (PV) of minced kilka during refrigerated storage. (control: without rosemary extract, FE 0.3%: 0.3% unencapsulated FE 0.5%: 0.5% unencapsulated fennel extract, E‐FE 0.3%: 0.3% encapsulated fennel extract, E‐FE 0.5%: 0.5% encapsulated fennel extract).

The effects of encapsulated and pure fennel extract on the changes in the PV content of the minced Kilka during 15 days storage at 4°C are shown in Figure 1. The peroxide value which detects a measure of the concentration of peroxides and hydroperoxides formed in the initial stages of lipid oxidation is widely used for the estimation of oxidative rancidity in fats (Ólafsdóttir et al. 1997). The PV values of samples significantly increased (*P *<* *0.05) during storage time, until day 9, and then decreased gradually until the end of storage time. After the maximum value was reached, a decrease in PV values was observed. This decrease is an unequivocal sign of advanced oxidation (Pereira de Abreu et al. 2010; Ariaii et al. 2014). A slower increase in PV values was obtained in samples treated with encapsulated and unencapsulated FE, in contrast to a faster increase in PV of the control sample after 3 days of storage time, demonstrating the oxidative stability of fish lipids by fennel extract. These results could be attributed to the antioxidant activity of fennel extract which is related to its polyphenol contents. This observation was in agreement with what reported by Chang et al. (2013) about the antioxidant properties of fennel extract. They showed that fennel extract had antioxidant effect on lipid stabilization by delaying the hydroperoxides formation.

At the end of storage time, the maximum and minimum PV were related to control (6.1 meq oxygen kg‐1 lipids) and 0.5% encapsulated FE (3.76 meq oxygen kg‐1 lipids), respectively. This indicated the potential of encapsulation to improve the antioxidant activity of the fennel extract during application in minced Kilka by prolonging its availability. Evidence of encapsulation improving the bioactivity and bioavailability of polyphenols has been reported by a number of researchers (Fang and Bhandari 2010). Furthermore, the results indicated that a high concentration of encapsulated FE was more effective in controlling oxidation. However, it has been reported that a higher concentration of extract may act as a better pro‐oxidant (Sarah et al. 2010).

### Changes in the total volatile basic nitrogen (TVB‐N)

**Figure 2:**
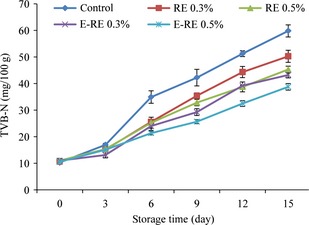
Changes in the total volatile basic nitrogen (TVB‐N) of minced kilka during refrigerated storage. (control: without rosemary extract, FE 0.3%: 0.3% unencapsulated fennel extract, FE 0.5%: 0.5% unencapsulated fennel extract, E‐FE 0.3%: 0.3% encapsulated fennel extract, E‐FE 0.5%: 0.5% encapsulated fennel extract).

The TVB‐N value is one of the most widely used indicators of seafood deterioration. It is a general term which includes the calculation of three methyl amine (produced by spoilage bacteria), dimethyl amine (produced by autolytic enzymes during chilled preservation), ammonia (produced by the deamination of amino acids and nucleotide catabolizes) as well as other volatile basic nitrogenous compounds correlated with seafood spoilage (Erikson et al. 2011). The effects of encapsulated and unencapsulated fennel extract on the changes in the TVB‐N content of the minced Kilka during 15 days storage at 4°C are shown in Figure 2. The initial TVB‐N value of the fillets was 10.66 mg/100 g which showed the good quality of fresh samples in that, freshwater fish muscle has 10–20 mg/100 g TVB‐N after harvesting (Alçiçek 2011). The value of TVB‐N increased progressively with the time of storage for all fish samples. TVB‐N increase in the control group was higher than fennel extract added groups during storage. After 3 days, the difference between the control group and the others was found to be significant (*P* < 0.05). At the end of storage time, the maximum and minimum TVB‐N were related to control (59.78 mg/100 g) and 0.5% encapsulated FE (38.76 mg/100 g), respectively. Since TVB‐N is produced mainly by bacterial decomposition and endogenous enzymes of fish muscle, the lower value of TVB‐N observed in samples treated with fennel extract can be related to the antibacterial activity of the extract which would more rapidly reduce the bacterial population and this could be due to the decreased capacity of bacteria for oxidative deamination of nonprotein nitrogen compounds (Banks et al. 1980). According to Connell (1990), a level of 35–40 mg TVB‐N/100 g of fish flesh is usually regarded as spoiled. The TVB‐N values of the samples in our study exceeded the maximum level by day 6 for control, by day 9 for 0.3% FE, and by day 12 for 0.5% FE and 0.3% encapsulated FE. It is lower than the acceptable limit for 0.5% encapsulated FE till the end of storage time. Anwar et al. (2009) reported appreciable antimicrobial activity for fennel extract and essential oils against selected strains of bacteria and pathogenic fungi. On the other hand, samples treated with gum arabic encapsulated fennel extract showed significantly lower TVB‐N content compared to control and fillets treated with pure extract during the storage period (*P *<* *0.05). This observation may be explained by the enhanced antimicrobial activity of the extract after encapsulation or the better protection of their functionality during the processing or storage period (Gortzi et al. 2007).

#### Microbiological changes

**Figure 3:**
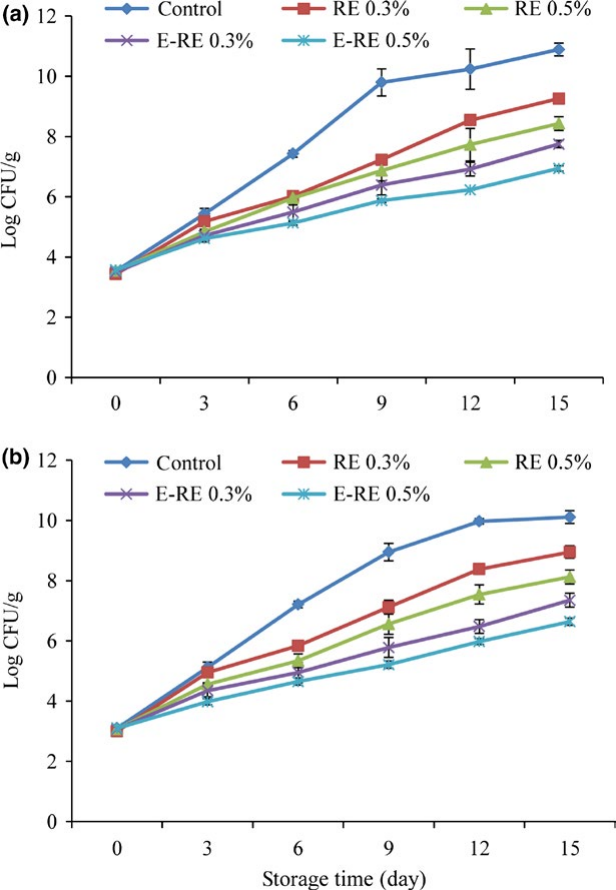
Changes in (A) total viable count (TVC) and (B) total psychrotrophic count (TPC) of minced kilka during refrigerated storage. (control: Without rosemary extract, FE 0.3%: 0.3% unencapsulated fennel extract, FE 0.5%: 0.5% unencapsulated fennel extract, E‐FE 0.3%: 0.3% encapsulated fennel extract, E‐FE 0.5%: 0.5% encapsulated fennel extract).

The composition of fish muscle makes it favorable for microbial growth. Thus, fish spoiling occurs during storage mainly as a result of microbial activity (Souza et al. 2010). Changes in the total viable count (TVC) and total psychrotrophic count (TPC) of minced Kilka are shown in Figure 3A and B. The initial TVC and TPC of fish sample was 3.5 and 3.1 log CFU/g, respectively, and the low initial TVC and TPC indicated very good fish quality (Fan et al. 2009).

Total viable count of all samples increased with storage time and the value of control increased faster and exceeded the maximum of 10^7^ log CFU/g after 6 day. This acceptability limit of 10^7^ CFU/g has been recommended for fresh fish (ICMSF, 1986). As can be seen, all minced samples treated with encapsulated and unencapsulated FE significantly inhibited the growth of mesophilic bacteria compared with the control during the storage period. These results showed antimicrobial properties of FE as a well known source of main component, such as contained (E)‐anethole, limonene, apiole, beta‐fenchyl acetate, and perillene known to exert antimicrobial activity (Cetin et al. 2010).

The total viable count of 0.5% encapsulated FE samples was lower than 7 CFU/g till the end of storage time. Significant changes were observed in microbial analysis of pure fennel samples and encapsulated fennel extract samples (*P *<* *0.05). A similar trend was also observed about TPC in all treatments. This indicated that encapsulated fennel extract could strongly inhibit the growth of TVC. The improvement of the antimicrobial activity of natural plant extracts and essential oils when encapsulated was also reported by others (Gortzi et al. 2006, 2007; Liolios et al. 2009; Donsì et al. 2011).

### Inhibition the growth of *Pseudomonas aeruginosa* in minced fish

**Figure 4:**
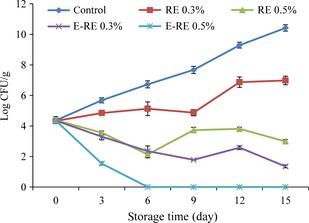
Changes in *Pseudomonas aeruginosa* count (Log CFU/g) in minced kilka during refrigerated storage. (control: Without rosemary extract, FE 0.3%: 0.3% unencapsulated fennel extract, FE 0.5%: 0.5% unencapsulated fennel extract, E‐FE 0.3%: 0.3% encapsulated fennel extract, E‐FE 0.5%: 0.5% encapsulated fennel extract).

The effects of encapsulated and unencapsulated fennel extract on *Pseudomonas aeruginosa* inoculated in Kilka mince are presented in Figure 4. As can be seen, the initial population of *Pseudomonas aeruginosa* in the control group increased rapidly during the storage period. Although, the sample treated with encapsulated and unencapsulated fennel extract showed that a population of *Pseudomonas aeruginosa* significantly (*P *<* *0.05) lowers than the control. Although the sample treated with unencapsulated FE at 0.3 showed that a population of *Pseudomonas aeruginosa* significantly lowers than the control, but it could not inhibit its growth completely during the storage period. Unencapsulated FE at 0.5% and encapsulated FE at 0.3% could significantly reduce the population of *Pseudomonas aeruginosa* compared to the control‐ and 0.3% FE‐treated samples. These results were in agreement with those reported by Careaga et al. (2003) about the effect of *Capsicum* extract on *Pseudomonas aeruginosa* inoculated in raw beef meet. They reported that raw beef samples treated with *Capsicum* extract at 0.3% exhibited the lower population of *Pseudomonas aeruginosa*, as compared with the untreated. Nevertheless, the drastic bactericidal effect of *Capsicum* extract was observed, when 4 or 5% extract was used, which was approximately 10‐fold more than the amount used in this study.

From day 6 until the end of storage, the degree of decline in the *Pseudomonas aeruginosa* population in 0.3% encapsulated FE samples was significantly (*P *<* *0.05) greater than in the unencapsulated FE 0.5% minced fish samples. According to the study, drastic bactericidal effect of fennel extract was observed in the encapsulated FE 0.5% minced fish samples during the mentioned days . These coincide with our observation about the TVC and TPC of minced Kilka which reveals the efficacy of encapsulation in improving the antimicrobial properties of FE.

## Conclusions

The effect of encapsultaed and unencapsulated fennel extract in controlling the quality of minced Kilka and *Pseudomonas aeruginosa* inoculated in the mince was studied. Results showed that fennel extract could efficiently retard the deterioration of Kilka minced, as reflected in lower TVB‐N and PV and microbial changes during the storage period. However, the encapsulated extract could act significantly better than unencapsulated fennel extract in all the studied properties, especially at 0.5% encapsulated FE. It was demonstrated that fennel extract was able to inhibit the growth of *P. aeruginosa*. The strong bactericidal effect of fennel extract was observed in the encapsulated FE 0.5%. The results obtained from this study support the idea of proposing the use of gum arabic encapsulation fennel extract as a natural antioxidant and antibacterial agent in an often contaminated food such as fish mince.

## Conflict of Interest

None declared.
